# A multicentric validation study of a novel home sleep apnea test based on peripheral arterial tonometry

**DOI:** 10.1093/sleep/zsac028

**Published:** 2022-02-02

**Authors:** Bart Van Pee, Frederik Massie, Steven Vits, Pauline Dreesen, Susie Klerkx, Jagdeep Bijwadia, Johan Verbraecken, Jeroen Bergmann

**Affiliations:** 1 Department of Engineering, Natural Interaction Lab, Thom Building, University of Oxford, Oxford, UK; 2 Faculty of Medicine and Health Sciences, University of Antwerp, Antwerp, Belgium; 3 Future Health Department, Ziekenhuis Oost-Limburg, Genk, Belgium and Mobile Health Unit, Faculty of Health and Life Sciences, Hasselt University, Hasselt, Belgium; 4 Department of Pneumology, Ziekenhuis Oost-Limburg, Genk, Belgium; 5 Department of Pulmonary Critical Care and Sleep Medicine, University of Minnesota, Minneapolis, MN, USA; 6 Department of Pulmonary Medicine and Multidisciplinary Sleep Disorders Centre, Antwerp University Hospital, Edegem,Belgium; 7 Research Group LEMP, Faculty of Medicine and Health Sciences, University of Antwerp, Antwerp, Belgium

**Keywords:** home sleep apnea testing, peripheral arterial tonometry, sympathetic tone, double-scoring, endpoint design

## Abstract

**Study Objectives:**

This paper reports on the multicentric validation of a novel FDA-cleared home sleep apnea test based on peripheral arterial tonometry (PAT HSAT).

**Methods:**

One hundred sixty-seven participants suspected of having obstructive sleep apnea (OSA) were included in a multicentric cohort. All patients underwent simultaneous polysomnography (PSG) and PAT HSAT, and all PSG data were independently double scored using both the recommended 1A rule for hypopnea, requiring a 3% desaturation or arousal (3% Rule), and the acceptable 1B rule for hypopnea, requiring a 4% desaturation (4% Rule). The double-scoring of PSG enabled a comparison of the agreement between PAT HSAT and PSG to the inter-rater agreement of PSG. Clinical endpoint parameters were selected to evaluate the device’s ability to determine the OSA severity category. Finally, a correction for near-boundary apnea–hypopnea index values was proposed to adequately handle the inter-rater variability of the PSG benchmark.

**Results:**

For both the 3% and the 4% Rules, most endpoint parameters showed a close agreement with PSG. The 4-way OSA severity categorization accuracy of PAT HSAT was strong, but nevertheless lower than the inter-rater agreement of PSG (70% vs 77% for the 3% Rule and 78% vs 81% for the 4% Rule).

**Conclusions:**

This paper reported on a multitude of robust endpoint parameters, in particular OSA severity categorization accuracies, while also benchmarking clinical performances against double-scored PSG. This study demonstrated strong agreement of PAT HSAT with PSG. The results of this study also suggest that different brands of PAT HSAT may have distinct clinical performance characteristics.

**Statement of Significance**

The paper reports on a recently FDA-cleared peripheral arterial tonometry (PAT)–based home sleep apnea testing device (HSAT), a category of HSAT devices that have seen a swift increase in clinical deployment owing to sleep clinic’s need for fully disposable devices.

Validation studies of HSATs have received scrutiny for their lack of robust clinical endpoint design and evaluation. This paper reports on a multitude of robust endpoint parameters, in particular, obstructive sleep apnea severity categorization accuracies, while also benchmarking clinical performances against double-scored polysomnography. The results also highlight that within the category of PAT HSATs, significant differences in accuracies may be found among the different embodiments.

## Introduction

The COVID-19 pandemic has reshaped how obstructive sleep apnea (OSA) diagnosis is being performed. Sleep labs face restrictions on the number of patients they can admit to their facilities during the outbreaks, which resulted in an accelerated shift from in-lab polysomnography (PSG) to home sleep apnea testing devices (HSATs). To mitigate the potential spread of infection, Ramar [1] stated how the field can benefit from the deployment of disposable HSAT. HSAT technology based on peripheral arterial tonometry (PAT) is especially well positioned to address this need as it is cost-effective to produce and can be deployed in a compact form, driving gains in logistics and ecological footprint.

### A brief history of peripheral arterial tonometry

In 1937, Hertzman published a paper titled “Photoelectric plethysmography of the Fingers and Toes in Man” [2], which would later be credited as the founding paper for the research into photoplethysmography (PPG) [3]. PPG operates based on optical technology to detect pulsatile blood volume changes in the tissue, from which blood oxygen level estimates can also be derived (i.e. pulse oximetry). Hertzman observed how changes in the tone of the peripheral arterial smooth muscle tissue, also referred to as peripheral arterial tone (PAT) and itself triggered by changes in sympathetic tone, were observable in the pulsatile blood volume changes as registered by the PPG. In a follow-up paper from 1942, Hertzman et al. [4] described the occurrence of such periodic changes in PAT in a snoring individual, in what we may today speculate to have been a patient with sleep apnea.

In the early 1970s, Lugaresi et al. [5] further complemented Hertzman’s work in his reporting of the simultaneous occurrence of respiratory disturbances with an increase in PAT, an acceleration in pulse rate, and the presence of a cortical arousal—an observation which closely resembles the AASM’s definition of the Peripheral Arterial Tonometry HSAT technique [6].

There are currently two FDA-cleared HSATs in the PAT category: WatchPAT (Itamar Medical, Israel) [7] and NightOwl (Ectosense, Belgium) [8], the latter of which is the device studied in this manuscript (Study Device). Both devices make use of signal conditioning methods to derive a sensitive PAT measurement from the PPG but differ in the mechanisms used to obtain such a measurement. WatchPAT uses mostly hardware implementations: an approximately isosbestic PPG wavelength provided through a third optical emitter helps compensate for fluctuations in the PPG driven solely by blood oxygen changes. A cuff-like pneumo-optic probe applies an approximately uniform and constant pressure with claims of improving the signal to noise ratio of the PPG as well as preventing venous pooling from affecting the PPG [9]. The Study Device comprises a wrap-around sensor probe—the size of a fingertip that does not fully envelop the finger. Instead, it relies mostly on signal processing techniques to compensate for varying levels of blood oxygen and the effects of venous pooling, as well as to obtain a highly linear measurement of PAT. These software-based techniques allow for an improved miniaturization of the technology.

### Study objective

The aim of this paper was to report on the multicentric validation of a novel home sleep apnea test based on peripheral arterial tonometry (PAT HSAT) with a particular emphasis on rethinking robust clinical endpoint design and evaluation.

## Methods

### Participants

One hundred sixty-seven participants suspected of having OSA were consecutively included in a cohort across four different centers of which one was located in Belgium (Ziekenhuis Oost Limburg, ZOL, Genk, Belgium) and three in the United States (where all centers were part of the United Health Systems Group in Miami, FL). All participants were scheduled for one overnight in-lab PSG. Participants were asked for informed consent. The US branch of the study was approved by Aspire Institutional Review Board (IRB), part of the WIRB-Copernicus Group. The European branch of the study was approved by the Ethics Committee of ZOL. Underaged or mentally impaired participants were excluded from participation in the study. For the European center, recruitment took place between July 2018 and September 2018. For the US-based centers, recruitment took place between December 2019 and January 2020. For all participants, gender, age, and Body-Mass-Index (BMI) were recorded. For the US branch of the study, participants completed the FDA’s self-completion questionnaire for ethnicity and race.

### Protocol and devices

**Figure 1.**
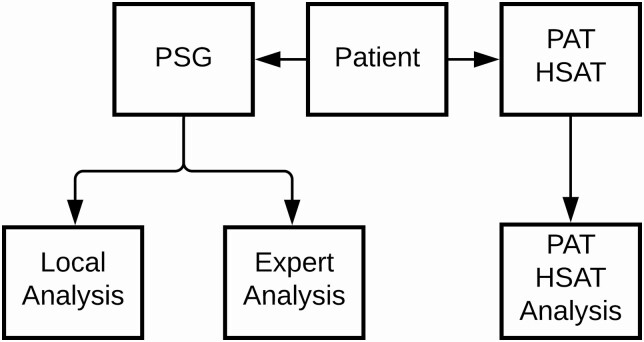
Diagram of the data acquisition setup. This diagram depicts that PSG and PAT HSAT were administered concurrently for each study participant. PSG was analyzed by two independent scoring centra (Expert and Local) and PAT HSAT was analyzed automatically. Both the Local Analysis and PAT HSAT Analysis were compared to Expert Analysis.

A graphical representation of the study setup is provided in Figure 1. Routine PSG was performed in all study participants. Qualified lab technicians at each participating study center were responsible for setting up the equipment and capturing PSG data. During the setup of PSG, the PAT HSAT (NightOwl, reusable version, software version 1.202.1) was attached to the middle finger of the hand to which the pulse oximeter of PSG was applied. All PSG data were double-scored by two independent centers which were blinded from one-another’s analysis.

### Polysomnography

For the European center, the Alice 6 PSG (Philips Respironics, USA) was used, whereas a Cadwell Easy PSG (Cadwell, USA) was applied in the US centers.

PSG was scored by two independent scoring centers. The first scoring was performed by the team of sleep technicians of the center where the patient was admitted (further referred to as the “Local Analysis”). A second independent scoring was performed by scorers of Cerebra Medical (CM, Canada), which provides computer-aided sleep scoring services to support PSG scoring for clinical centers and clinical trials. The studies were first analyzed by their proprietary Michele Sleep Scoring System (MSSS) and were subsequently complemented with complete manual rescoring by an expert technologist. All expert scorers of CM had received Registered Polysomnographic Technologist certification through the Board of Registered Polysomnographic Technologists.

Malhotra et al. [10] confirmed in a multicentric trial that the MSSS, complemented with manual editing by an expert scorer, is more robust than the results of a single expert scorer. Because of these conclusions, CM’s analysis served as the expert benchmark to which the Local Analysis and PAT HSAT’s analysis were compared. The analysis by CM is therefore referred to as the “Expert Analysis.”

All PSG data were scored according to the latest AASM scoring rules [6]. Data were first scored using the *Recommended rule 1A* for the scoring of hypopnea (3% Rule), requiring a 3% desaturation or an arousal for the scoring of hypopnea. An alternative scoring of all PSGs was also performed using the *Acceptable rule 1B* (4% Rule) for the scoring of hypopnea by discarding all hypopnea that did not coincide with an oxygen desaturation of at least 4%.

## Statistical Analysis

### General

Statistical analysis was performed using MATLAB (version 2019a, MathWorks, USA). For all endpoint parameters, 95% confidence intervals were computed. For proportion-based endpoints, confidence intervals were computed by approximating the distribution of error about a binomially distributed observation with a normal distribution. Significance levels were set at a p-value of .05. The PAT HSAT outcome was compared to the Expert Analysis for both the 3% Rule and the 4% Rule by using the PAT HSAT pAHI as scored by its 3% Rule and 4% Rule scoring variant. Significant differences between two proportions were assessed by means of a *two-proportion z-test.*

### Data synchronization

PSG and PAT HSAT data were algorithmically synchronized by matching the instantaneous heart rate traces derived from the electrocardiogram trace of PSG and the PR trace of PAT HSAT. Data epochs that were of insufficient quality to be interpreted by the sleep technician or PAT HSAT were rejected from both PSG and PAT HSAT traces. This resulted in a median rejection rate of 12% of data epochs per recording.

### Data adequacy

#### Technical failure of PSG and missing PSG data or annotations.

The AASM defines HSAT as technically adequate if at least 4 h of analyzable signal can be obtained [11]. In our study, the same cutoff criterion of technical adequacy was used, and all technically inadequate recordings were excluded from further analysis. When PSG recording was technically inadequate, for instance, when one of the channels could not be interpreted by the technicians, the participant was excluded from analysis. Similarly, when PSG data or any annotations of the two scoring centers were missing due to administrative errors, the participant was removed from further analysis.

Participants with missing patient characteristics, such as age and gender data, were omitted from the analysis of population demographic statistics.

### Performance endpoint selection

The clinical performance of HSATs can be described by their (diagnostic) accuracy, defined as the percentage agreement with polysomnography of the obstructive sleep apnea severity category (normal, mild, moderate, and severe) [12].

Secondary performance endpoints which characterize the device’s bias and variance in estimating the apnea–hypopnea index (AHI) may provide additional insights as to the device’s propensity to over-or underestimate the OSA severity. In light of the considerations mentioned above, a list of primary and secondary endpoints is proposed.

### Primary endpoints

#### OSA severity categorization accuracy (4-way categorization accuracy)

The 4-way categorization accuracy expresses the percentage of agreement between the OSA severity determined by HSAT and the OSA severity determined by PSG. Its main advantage is its straightforward interpretation. Its main disadvantage is its lack of insight into whether the categorization performance of HSAT exceeds the agreement that can be obtained by random guessing (chance level). Consider an extreme example where 90% of the study participants have mild OSA. In such a case, it is trivial for HSAT to obtain a 90% categorization accuracy by outputting mild OSA 100% of the time without performing any meaningful inference. The 4-way accuracy of 90% would misleadingly suggest that HSAT is effective.

#### *Cohen’s Kappa* (κ)

To address the main limitation of categorization accuracies, Cohen’s Kappa [13] is an alternative agreement metric which takes into account the chance level. Cohen’s Kappa is formulated as follows:


$$\begin{array}{l} \kappa =\frac{categorization\;\;\;accuracy-chance\;\;\;level}{1-chance\;\;\;level} \end{array}$$

The downside of this metric is its less straightforward interpretation. Applying this formula to the previous example, Cohen’s Kappa corresponding to the 90% categorization accuracy would be 0.

#### Confusion matrix, sensitivity (Se), specificity (Sp), negative predictive value (NPV), positive predictive value (PPV), and cutoff agreement (Acc)

Confusion matrices and their derived parameters provide additional granularity to the categorization accuracy and Cohen’s Kappa since they expose whether HSAT tends to over- or underestimate certain OSA severity categories.

### Secondary endpoints

#### Bland–Altman analysis

In order to describe the bias and variance of the AHI estimates, a Bland–Altman analysis can be performed, which sets out the average AHI of the reference and comparator against their difference. The standard Bland–Altman analysis is sensitive to extreme values, typically occurring at higher AHIs. Therefore, we propose to complement the standard Bland–Altman analysis with a sub-analysis in which only reference AHIs smaller than 30 are retained. For non-normally distributed differences, the limits of agreement (LoA) were determined as the 97.5th and 2.5th percentiles of the differences. For normally distributed differences, the LoA were determined as the mean ±1.96 times the standard deviation of the residuals. We also performed a Bland–Altman analysis for the (estimated) total sleep time (TST).

#### ICC(A,1)

The degree of absolute agreement between two AHI estimates (and other parameters such as TST) can be described by the intraclass correlation coefficient of the type *two-way fixed model with single measures of absolute agreement* (ICC(A,1)) [14].

In a context where absolute agreement rather than merely a linear relationship is important, the ICC(A,1) is a more robust and targeted parameter than the commonly used Pearson or Spearman correlation coefficient. The Pearson correlation coefficient attains the maximum value of 1 upon a perfect linear relationship between the two raters’ observations, but it does not penalize a constant offset or a scaling factor between them. For example, if the AHI determined by HSAT would be consistently equal to twice the AHI of the PSG increased by 10 events per hour, a perfect linear relationship would exist, and the Pearson correlation coefficient would attain the maximum value of 1. Nevertheless, such HSAT would have impaired clinical utility. Worse in this context, is the Spearman correlation coefficient, as it attains the maximum value of 1 when there is a perfect monotonously increasing relationship between the two variables without penalizing for non-linearity of such relationship [15]. When absolute agreement needs to be assessed, these coefficients provide misleadingly high values for HSAT and should be avoided [15]. The ICC(A,1) does penalize both issues and attains the maximum value of 1 only upon a perfect match (i.e. absolute agreement) between the raters’ observations. Nevertheless, the ICC(A,1), similar to most other correlation coefficients, is heavily influenced by outliers. Therefore, to assess this influence, we included an additional ICC(A,1) which was calculated on only those participants for which the Expert Analysis’ AHI was less than 30. Confidence intervals for the ICC(A,1) were calculated as described by McGraw et al [14].

### Endpoint assessment

No consensus exists on what endpoint parameter values are required to permit the conclusion that a HSAT has adequate performance. In order to avoid the creation of arbitrary endpoint targets, we compared each endpoint parameter calculated from the HSAT to PSG comparison to the same endpoint parameter calculated from two independent scorings of the same PSG to which HSAT is compared. For this study, we compared the endpoint parameters calculated from comparing the PAT HSAT analysis to the Expert PSG Analysis to those calculated from comparing the Local PSG analysis to the Expert PSG Analysis. For all endpoint parameters, we then assessed whether its value for the HSAT-PSG comparison was significantly less favorable than the PSG scorer-to-scorer comparison.

### Handling AHIs close to OSA severity category boundaries

Significant inter-rater disagreement on key diagnostic parameters such as the AHI exists [10]. This implies that an AHI derived by PSG that is close to any of the OSA severity category boundaries (5, 15, and 30) should be treated with caution. For example, an AHI of 15.1 would qualify as moderate, whereas an AHI of 14.9 would qualify as mild, which could have different therapeutic implications. However, this difference in AHI is much smaller than the typical inter-rater variability of the AHI. A dataset in which a significant proportion of AHIs are close to the OSA severity boundaries (near-boundary AHIs) could provide an overly pessimistic assessment of HSAT performance. Therefore, we complemented any endpoint analysis based on AHI cutoffs with an alternative endpoint parameter calculation that corrects for near-boundary AHIs. Concretely, we allocated two possible OSA severity categories to near-boundary Expert Analysis’ AHIs. This process was called near-boundary double-labeling (NBL). For example, an Expert Analysis’ AHI of 14.9 would receive the label of *mild OR moderate OSA* rather than just *mild OSA*. As a result, if a HSAT detects *moderate OSA*, this scoring should be considered in agreement with the Expert Analysis. For endpoint parameters that are evaluated at a single AHI cutoff, the same NBL principle can be applied. For example, if the agreement at AHI cutoff 15 is evaluated and if the Expert Analysis’ AHI is very close to 15, the ground truth AHI severity category is similarly likely to be either mild or moderate and is as such to be considered in default agreement with HSAT’s or Local Analysis’ AHI categorization at cutoff 15.

When implementing boundary corrections, it is important to establish adequate ranges for near-boundary-zones (NBZ), for which Expert Analysis’ AHIs falling within should receive NBL. We determined the NBZ from analyzing the double-scored PSG data obtained in this study.

In a first step, we estimated the probability that a second scoring of the PSG data would end up in a different OSA severity category from the Expert Analysis (OSA severity disagreement probability). For each AHI value ranging from 0 to 40 (the reference AHI), evaluated at increments of 0.2 events per hour, we gathered the observed AHI differences of the two PSG scorings (i.e. the Expert and Local scorings) for which one of the two scorer’s AHI was within a range of 5 events per hour of that reference AHI. We included those AHI differences in what was named the nearby sample set for that particular reference AHI. We could then fit a normal distribution onto the nearby sample set of each reference AHI by taking the reference AHI as the mean of the distribution with a standard deviation equal to that found within the nearby sample set of AHI differences. From the resulting AHI disagreement probability distribution, we could straightforwardly calculate, for each reference AHI, the OSA severity disagreement probability. The range of 5 to determine the nearby sample set was not reduced to a narrower range as this would require a larger dataset to maintain the smoothness of the OSA severity disagreement probability curve (expressed as the number of slope sign changes of the curve). Normality of the nearby sample set was evaluated by means of the Anderson–Darling test. For the purpose of this study, we defined the NBZ as those AHI ranges for which there is a one out of three (33%) OSA severity disagreement probability. The reason for this cutoff choice is twofold. Although this cut-off may differ with individual practitioners’ preferences, only double-labeling those AHI values where the OSA severity disagreement probability is 50% would result in no double labeling, as only those reference values with exactly the boundary cut-offs (5.0, 15.0, and 30.0) would be double-labeled. Conversely, an OSA severity disagreement probability cut-off of 15% on this dataset would double-label all AHIs except for very severe OSA patients.

### Sample size determination

Statistical power was determined by postulating that a 10% decrease in OSA severity categorization accuracy should be detected as statistically significant with an alpha 0.05 and a power of 0.8. Assuming a minimum 4-way categorization accuracy parameter value of 0.75 for the Local Analysis, a minimum sample size of 165 participants was found.

## Results

### Participant inclusion and population statistics

**Figure 2.**
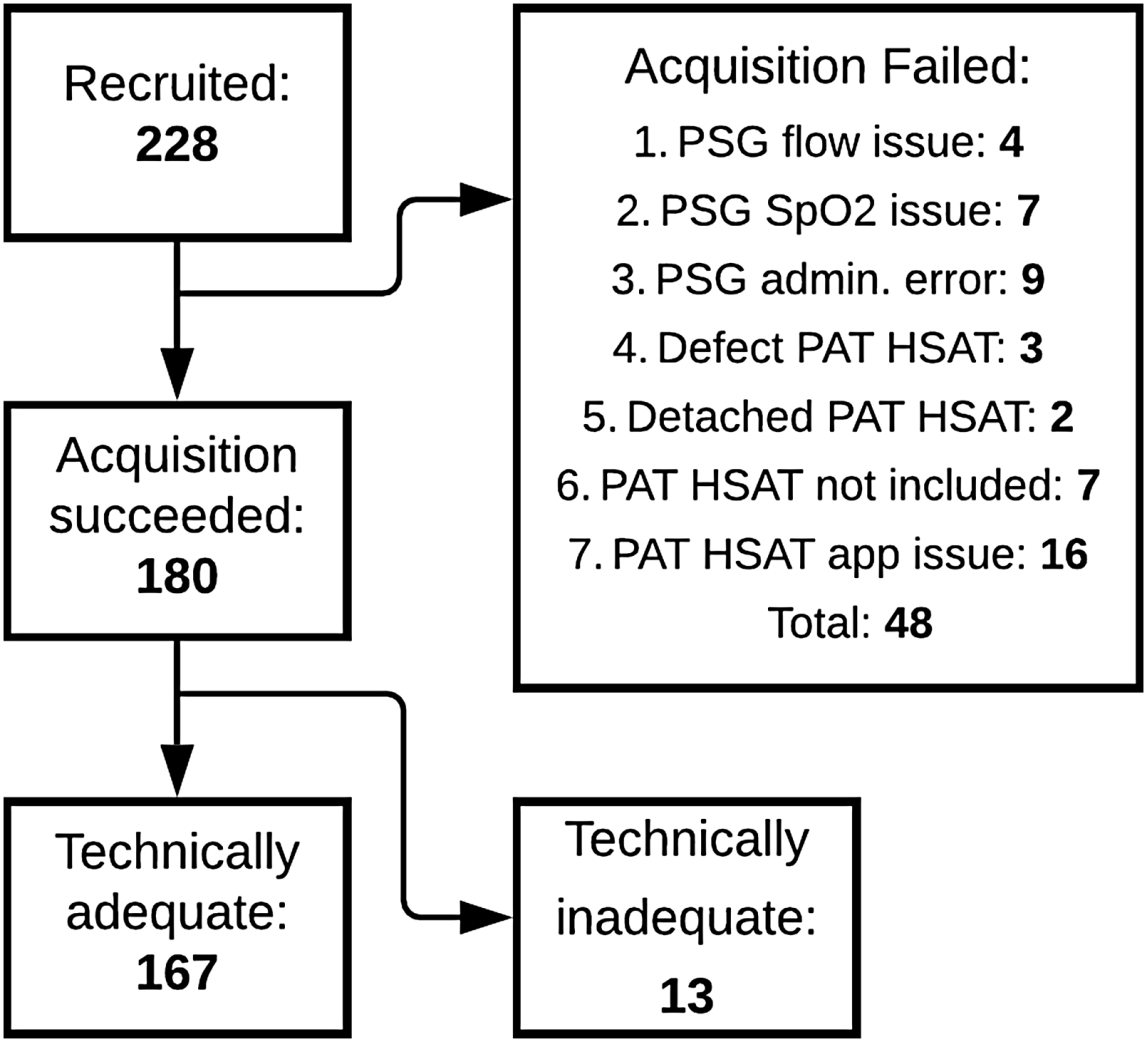
Flowchart of participant recruitment. This flowchart depicts how many participants gave informed consent (228), for how many participants PSG and PAT HSAT data acquisition was successful (180), and for how many participants the PAT HSAT was technically adequate (> 4 h of interpretable data). Additionally, the flowchart summarizes the reasons for data acquisition failure. SpO_2_ = blood oxygen saturation.

Figure 2 provides a flowchart highlighting the number of recruited participants as well as administrative and technical failures, including the reason for failure. Out of the 228 participants who gave informed consent, concurrent PSG and PAT HSAT data were successfully acquired for 180 participants. For respectively 4 and 7 participants, there was an issue with the flow or SpO_2_ channel of the PSG, rendering scoring impossible. For 9 participants, PSG was only single-scored or the link between the PAT HSAT and PSG could no longer be retrieved. Three participants received a defective PAT HSAT sensor that did not acquire any data. Two participants detached the PAT HSAT sensor early in the study. For 7 participants who gave informed consent, eventually no PAT HSAT data acquisition was started. For 16 participants in the European branch of the study, an early prototype version of the PAT HSAT data acquisition app experienced stability issues resulting in a loss of data. For 13 out of the 180 successful inclusions, the PAT HSAT recordings were not considered technically adequate since less than 4 h of interpretable data could be acquired. As a result, technically adequate data were acquired for 167 participants. Of these 167 technically adequate inclusions, 74 were performed in the United States and 93 in Europe. As elaborated in Table 1, participants were predominantly male (63%), of middle age (mean 56 years, STD 15), and overweight (mean BMI 30.7 and STD 6.3). The mean AHI was 32.7 (STD 26.8). Twenty-two participants had no OSA, 37 participants had mild OSA, 33 participants had moderate OSA, and 75 participants had severe OSA.

**Table 1.** Demographic and clinical characteristics of participants in dataset

	Mean	STD	Min	Max
Age	56	15	21	84
BMI	30.7	6.3	18.2	53.8
AHI	32.7	26.8	0.0	114.9
	*N* (%)			
No OSA	22(13%)			
Mild OSA	37(22%)			
Moderate OSA	33(20%)			
Severe OSA	75(45%)			
Black	43 (26%)			
White	124 (74%)			
Hispanic, Latino, or Spanish	92 (55%)			

AHI = apnea–hypopnea index; BMI = body mass index; *N* = number of participants; OSA = obstructive sleep apnea; STD = standard deviation.

In the US branch of the study, 26% of participants identified as black, 74% as white, and 55% as Hispanic, Latino, or Spanish.

### Near-boundary determination

**Figure 3.**
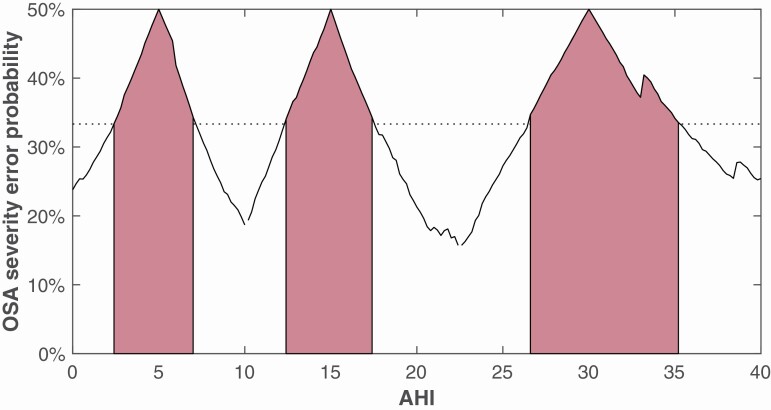
OSA severity error probability. This figure displays for each AHI the probability that a second PSG scoring would result in an AHI with a different OSA severity category. The red zones highlight those AHI values for which this probability is larger than 33% (dotted line).

Figure 3 displays the results of the near-boundary AHI determination is described in the Methods section. The figure displays for each AHI the OSA severity disagreement probability. The red zones highlight those AHI values for which this probability is larger than 33%. As such, these near-boundary zones can be summarized as displayed in Table 2.

**Table 2.** OSA severity categories when applying near-boundary double-labeling

OSA severity categories using NBL	AHI Range	*N* (%)
Normal	0.0 ≤ AHI < 2.4	12 (7.2%)
*Normal OR Mild (NBZ)*	2.4 ≤ AHI < 7.0	21 (12.6%)
Mild	7.0 ≤ AHI < 12.4	17 (10.2%)
*Mild OR Moderate (NBZ)*	12.4 ≤ AHI < 17.4	15 (9.0%)
Moderate	17.4 ≤ AHI < 26.6	21 (12.6%)
*Moderate OR Severe (NBZ)*	26.6 ≤ AHI < 35.2	14 (8.4%)
Severe	35.2 ≤ AHI	67 (40.0%)

The near-boundary zone is defined as the zone for which the probability that a second scoring of the AHI would fall in a different OSA severity category exceeds 33%. NBZ are highlighted in italics.

AHI = apnea–hypopnea index; *N* = number of paricipants within each category (according to Expert Analysis); NBL = near-boundary double-labeling; NBZ = near-boundary zones; OSA = obstructive sleep apnea.

The values presented in this table were used when calculating primary endpoint parameters using near-boundary double-labeling. For AHIs ranging from 0 to 12 and from 33 to 40, the normal distribution assumption of the nearby sample set was not met according to the Anderson–Darling test. A violation of this assumption might render the error probability estimates less accurate in those regions.

### Primary endpoint parameters

Table 3 displays all primary endpoint parameters calculated for PAT HSAT as well as the Local PSG Analysis, for both the 3% Rule and 4% Rule, and for both the regular endpoint parameter calculation and the alternative calculation with NBL. Whenever PAT HSAT or Local Analysis significantly outperformed one another, the outperforming endpoint parameter is highlighted with an asterisk.

**Table 3.** Endpoint parameter result summary table

Endpoint parameter		NBL	3% Rule		4% Rule	
			PAT HSAT	Local PSG	PAT HSAT	Local PSG
4-way categorization accuracy		*off*	0.70 (0.63–0.77)	0.77 (0.70–0.83)	0.78 (0.72–0.84)	0.81 (0.76–0.87)
		*on*	0.81 (0.75–0.87)	0.88 (0.83–0.93)*	0.89 (0.84–0.93)	0.89 (0.84–0.93)
4-way Cohen’s Kappa		*off*	0.56 (0.46–0.66)	0.66 (0.57–0.76)*	0.70 (0.62–0.79)	0.75 (0.67–0.83)
		*on*	0.72 (0.64–0.81)	0.83 (0.76–0.90)*	0.85 (0.78–0.91)	0.85 (0.78–0.91)
Cutoff AHI 5	Se	*off*	0.93 (0.89–0.97)	0.95 (0.93–0.99)	0.95 (0.91–0.99)	0.97 (0.94–1.00)
		*on*	0.97 (0.94–1.00)	0.99 (0.98–1.00)	0.97 (0.94–1.00)	0.98 (0.96–1.00)
	Sp	*off*	0.72 (0.54–0.91)	0.72 (0.54–0.91)	0.80 (0.68–0.92)	0.84 (0.73–0.95)
		*on*	0.85 (0.71–0.95)	0.88 (0.74–1.00)	1.00 (0.96–1.00)*	0.88 (0.79–0.98)
	NPV	*off*	0.62 (0.42–0.80)	0.73 (0.54–0.91)	0.85 (0.75–0.96)	0.90 (0.81–0.99)
		*on*	0.85 (0.71–0.99)	0.95 (0.87–1.00)	0.90 (0.81–0.99)	0.95 (0.88–1.00)
	PPV	*off*	0.96 (0.92–0.99)	0.96 (0.93–0.99)	0.93 (0.88–0.97)	0.94 (0.90–0.98)
		*on*	0.97 (0.94–1.00)	0.98 (0.96–1.00)	1.00 (0.97–1.00)	0.99 (0.93–1.00)
	Acc	*off*	0.90 (0.86–0.95)	0.93 (0.89–0.97)	0.91 (0.87–0.95)	0.93 (0.90–0.97)
		*on*	0.95 (0.92–0.98)	0.98 (0.95–1.00)	0.98 (0.95–1.00)	0.96 (0.93–0.99)
	Kappa	*off*	0.60 (0.43–0.79)	0.69 (0.52–0.86)	0.76 (0.65–0.88)	0.83 (0.73–0.93)
		*on*	0.82 (0.70–0.94)	0.90 (0.80–1.00)*	0.93 (0.87–1.00)	0.89 (0.81–0.97)
Cutoff AHI 15	Se	*off*	0.91 (0.85–0.96)	0.94 (0.89–0.98)	0.94 (0.90–0.99)	0.94 (0.90–0.99)
		*on*	0.94 (0.89–0.98)	0.95 (0.92–0.99)	0.96 (0.92–1.00)	0.97 (0.93–1.00)
	Sp	*off*	0.76 (0.65–0.87)	0.85 (0.76–0.94)	0.91 (0.85–0.97)	0.86 (0.78–0.94)
		*on*	0.83 (0.73–0.93)	0.90 (0.82–0.98)	0.95 (0.90–1.00)	0.90 (0.83–0.96)
	NPV	*off*	0.82 (0.72–0.92)	0.87 (0.79–0.96)	0.93 (0.88–0.99)	0.93 (0.87–0.99)
		*on*	0.87 (0.79–0.96)	0.91 (0.84–0.99)	0.95 (0.90–1.00)	0.96 (0.91–1.00)
	PPV	*off*	0.88 (0.81–0.94)	0.92 (0.87–0.97)	0.92 (0.87–0.98)	0.89 (0.82–0.95)
		*on*	0.91 (0.86–0.96)	0.95 (0.90–0.99)	0.96 (0.92–1.00)	0.92 (0.86–0.97)
	Acc	*off*	0.86 (0.80–0.91)	0.90 (0.86–0.95)	0.93 (0.89–0.97)	0.90 (0.86–0.95)
		*on*	0.90 (0.85–0.94)	0.93 (0.90–0.97)	0.95 (0.92–0.99)	0.93 (0.90–0.97)
	Kappa	*off*	0.68 (0.56–0.80)	0.79 (0.69–0.89)*	0.86 (0.78–0.93)	0.81 (0.72–0.90)
		*on*	0.77 (0.67–0.88)	0.85 (0.77–0.94)*	0.90 (0.84–0.97)	0.87 (0.79–0.94)
Cutoff AHI 30	Se	*off*	0.91 (0.84–0.97)	0.91 (0.84–0.97)	0.86 (0.77–0.95)	0.95 (0.89–1.00)
		*on*	0.93 (0.88–0.99)	0.95 (0.90–1.00)	0.91 (0.83–0.98)	1.00 (0.96–1.00)*
	Sp	*off*	0.91 (0.86–0.97)	0.92 (0.87–0.98)	0.96 (0.93–1.00)	0.95 (0.90–0.99)
		*on*	0.94 (0.88–0.99)	0.96 (0.92–1.00)	0.97 (0.93–1.00)	0.95 (0.91–0.99)
	NPV	*off*	0.92 (0.87–0.98)	0.92 (0.87–0.98)	0.93 (0.88–0.98)	0.97 (0.94–1.00)
		*on*	0.95 (0.90–0.99)	0.96 (0.92–1.00)	0.96 (0.92–0.99)	1.00 (0.97–1.00)
	PPV	*off*	0.90 (0.83–0.96)	0.91 (0.84–0.97)	0.92 (0.85–1.00)	0.90 (0.82–0.98)
		*on*	0.92 (0.86–0.98)	0.95 (0.90–1.00)	0.92 (0.85–1.00)	0.90 (0.82–0.98)
	Acc	*off*	0.91 (0.87–0.95)	0.92 (0.87–0.96)	0.93 (0.89–0.97)	0.91 (0.91–0.98)
		*on*	0.93 (0.90–0.97)	0.95 (0.92–0.99)	0.95 (0.91–0.98)	0.96 (0.94–0.99)
	Kappa	*off*	0.82 (0.73–0.91)	0.83 (0.75–0.92)	0.84 (0.75–0.93)	0.88 (0.81–0.96)
		*on*	0.87 (0.79–0.94)	0.90 (0.83–0.97)	0.88 (0.80–0.95)	0.92 (0.86–0.98)

This table displays all endpoint parameter values and their confidence intervals (between brackets) for PAT HSAT and the Local PSG and for the 3% rule and the 4% rule, with and without NBL. An asterisk signifies a significant outperformance of the endpoint parameter (within the same scoring rule selection). Acc = accuracy; AHI = apnea–hypopnea index; Kappa = Cohen’s Kappa; NBL = near-boundary double-labeling; NPV = negative predictive value; PAT HSAT = home sleep apnea test based on peripheral arterial tonometry; PPV = positive predictive value; PSG = polysomnography; Se = sensitivity; Sp = specificity.

The 4-class accuracy using NBL as well as the Cohen’s Kappa (with and without NBL) was significantly lower for PAT HSAT compared to the Local analysis for the 3% Rule. The specificity at AHI cutoff 5 after NBL was significantly higher for PAT HSAT for the 4% Rule. The Cohen’s Kappa for the same cutoff after NBL was significantly lower for PAT HSAT for the 3% Rule. Another significant underperformance of PAT HSAT was found for AHI cutoff 15 for the calculation of Cohen’s Kappa both with and without NBL. Finally, the sensitivity of the Local Analysis at AHI cutoff 30 calculated with NBL was significantly higher than PAT HSAT’s for the 4% rule. No other statistically significant differences were found.

The impact of near-boundary AHIs on performance calculations becomes apparent from the performance gain reported in all endpoint parameters when applying NBL. For 84% of all reported endpoint parameter values, the value for the 4% Rule was higher than or equal to that of the 3% Rule. Table 4 displays the confusion matrices for the OSA severity of PAT HSAT compared to the Expert Analysis as well as for the Local Analysis compared to the Expert Analysis. The confusion matrices were generated for the 3% Rule and the 4% Rule as well as for OSA severity categorization with and without application of NBL, resulting in four different confusion matrices.

**Table 4.** OSA severity category confusion matrix

		PAT HSAT | Local PSG—3% Rule				PAT HSAT | Local PSG—4% Rule			
		No	Mild	Mod.	Severe	No	Mild	Mod.	Severe
**Expert PSG** | NBL off	No	**16** | 16	**4** | 5	**2** | 1	**0** | 0	**35** | 37	**9** | 3	**0** | 4	**0** | 0
	Mild	**9** | 5	**16** | 24	**12** | 8	**0** | 0	**5** | 3	**21** | 23	**7** | 7	**0** | 0
	Mod.	**1** | 0	**8** | 6	**16** | 20	**8** | 7	**1** | 1	**3** | 4	**26** | 23	**4** | 6
	Severe	**0** | 1	**1** | 0	**6** | 6	**68** | 68	**0** | 0	**1** | 0	**7** | 3	**48** | 53
**Expert PSG** | NBL on	No	**22** | 21	**2** | 2	**2** | 1	**0** | 0	**37** | 39	**0** | 1	**0** | 4	**0** | 0
	Mild	**3** | 0	**21** | 29	**8** | 5	**0** | 0	**3** | 1	**31** | 27	**4** | 4	**0** | 0
	Mod.	**1** | 0	**5** | 4	**22** | 26	**6** | 4	**1** | 1	**2** | 2	**32** | 29	**4** | 6
	Severe	**0** | 1	**1** | 0	**4** | 3	**70** | 71	**0** | 0	**1** | 0	**4** | 0	**48** | 53

This table displays the confusion matrix comparing the OSA severity category as determined by the Expert PSG analysis to respectively PAT HSAT (bold font) and Local PSG analysis (normal font) for the 3% rule (L) and the 4% rule (R), with NBL (Bottom) and without NBL (Top). Mod. = moderate sleep apnea; NBL = near-boundary double-labeling; OSA = obstructive sleep apnea; PAT HSAT = home sleep apnea test based on peripheral arterial tonometry; PSG = polysomnography.

### Secondary endpoint parameters

#### AHI AHI

**Figure 4.**
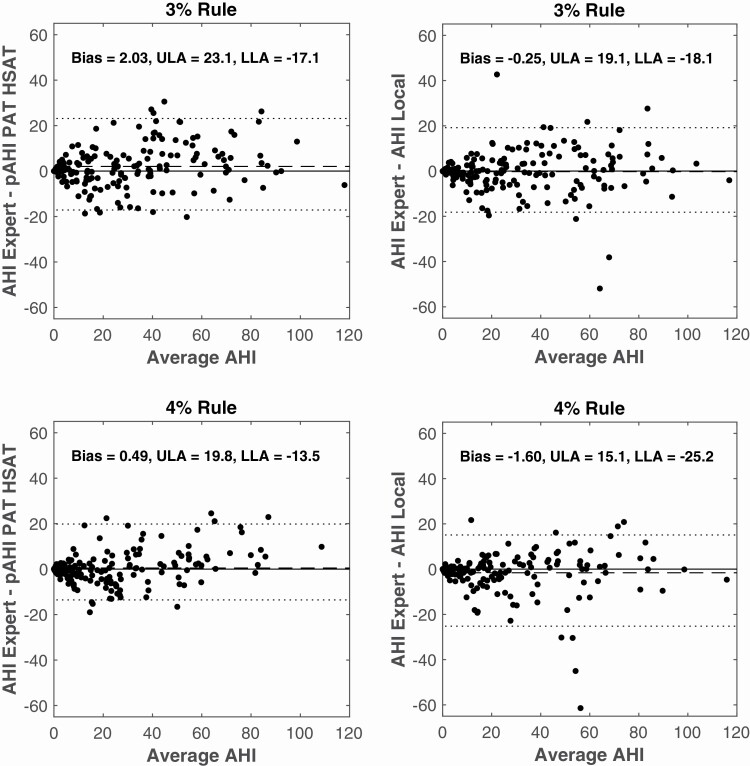
Bland–Altman plots of AHI determined by PAT HSAT (L) and Local Analysis (R) versus Expert Analysis for the 3% rule (Top) and the 4% rule (Bottom). The dotted line represents the mean Limits of Agreement. The dashed line represents the mean difference or bias. Average AHI = average of AHI Expert and pAHI PAT HSAT (L) or AHI Expert and AHI Local (R); LLA = lower limit of agreement; pAHI = AHI estimated by peripheral arterial tonometry.

**Figure 5.**
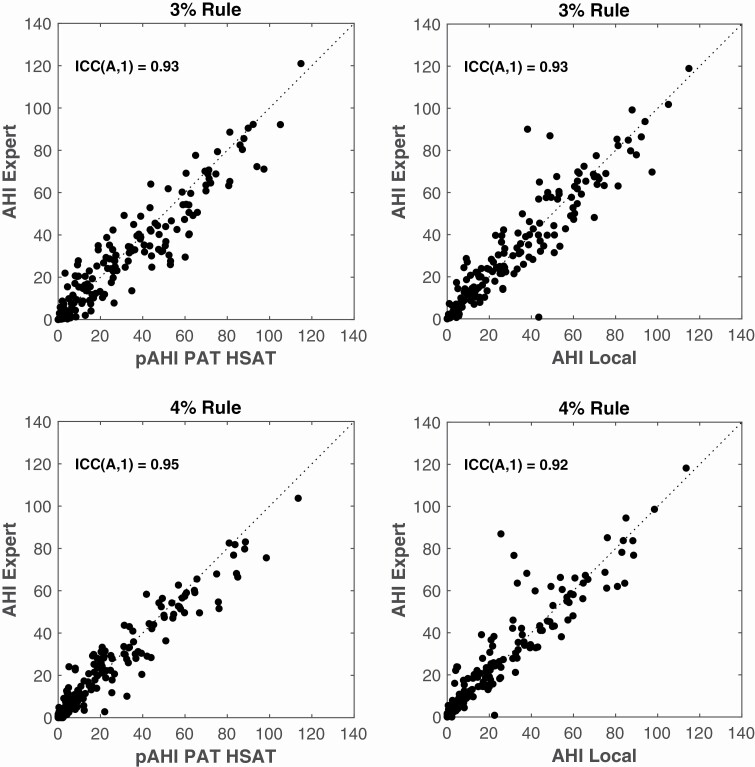
Scatter plots of AHI determined by PAT HSAT (L) and Local Analysis (R) versus Expert Analysis for the 3% rule (Top) and the 4% rule (Bottom). The dotted line represents the points for which the y-axis values equal the x-axis values of the graph (identity line). ICC(A,1) = Intraclass correlation coefficient of type (A,1); pAHI = AHI estimated by peripheral arterial tonometry.

Figures 4 and 5 as well as Table 5 show the results of a Bland–Altman and correlation analysis for both scoring rules. A significantly higher ICC(A,1) was found for PAT HSAT compared to the Local PSG for the 4% Rule. Removing AHIs larger than 30 significantly reduced the ICC(A,1) for both PAT HSAT and Local Analysis, which highlights the misleading influence of extreme values on this parameter. The width of the limits of agreement significantly reduced after removing AHIs larger than 30.

**Table 5.** Summary of Bland–Altman and correlation analysis of AHI

		3% Rule		4% Rule	
		PAT HSAT	Local PSG	PAT HSAT	Local PSG
**All AHIs**	Bias	2.03	−0.35	0.49	−1.60
	ULA	23.1	19.1	19.8	15.1
	LLA	−17.1	−18.1	−13.5	−25.2
	ICC(A,1)	0.93 (0.90–0.95)	0.93 (0.91–0.95)	0.95 (0.94–0.96)*	0.93 (0.90–0.95)
**AHIs < 30**	Bias	−1.29	−1.81	−1.77	−2.40
	ULA	10.9	8.6	8.5	6.6
	LLA	−17.0	−16.8	−14.1	−19.1
	ICC(A,1)	0.74 (0.63–0.82)	0.79 (0.69–0.86)	0.81 (0.72–0.87)*	0.68 (0.55–0.77)

This table displays the bias, ULA, LLA, and ICC(A,1) from the Bland–Altman and correlation analysis comparing the pAHI of PAT HSAT and the AHI of the Local PSG Analysis to the Expert PSG Analysis. When confidence intervals were calculated they are displayed between brackets. An asterisk signifies a significant outperformance of the endpoint parameter (within the same scoring rule selection). These parameters are calculated for the 3% Rule and the 4% Rule and for all AHIs as well as only AHIs < 30 (as determined by Expert PSG Analysis). AHI = apnea–hypopnea index; ICC(A,1) = intraclass correlation coefficient of type (A,1); LLA = lower limits of agreement; pAHI = AHI estimated by peripheral arterial tonometry; PAT HSAT = home sleep apnea test based on peripheral arterial tonometry; PSG = polysomnography; ULA = upper limits of agreement.

#### TST

**Figure 6.**
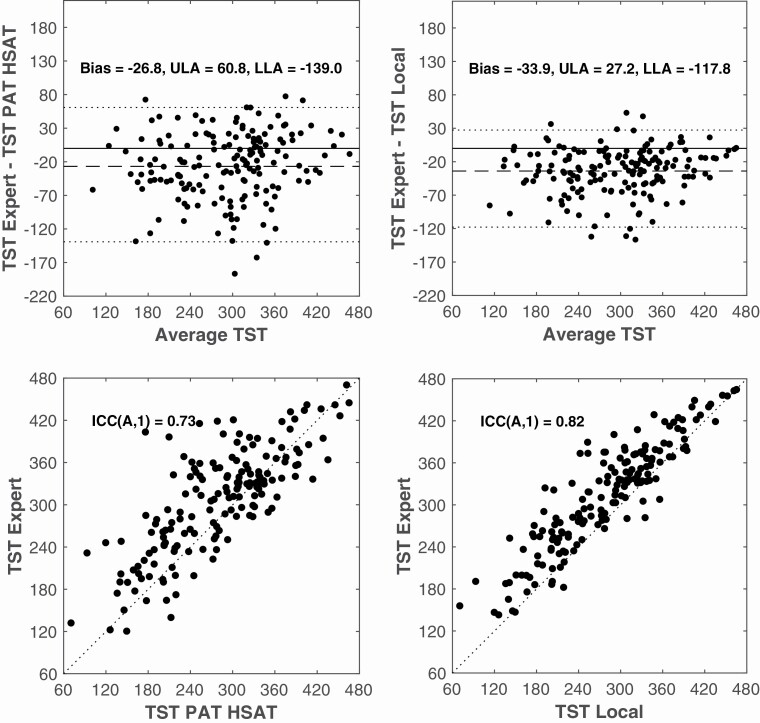
Bland–Altman (Top) and Scatter (Bottom) plots of TST determined by PAT HSAT (L) and Local Analysis (R) versus Expert Analysis. Top: the dotted line represents the mean Limits of Agreement. The dashed line represents the mean difference or bias. Bottom: the dotted line represents the points for which the y-axis values equal the x-axis values of the graph (identity line). Average TST = average of TST Expert and TST PAT HSAT (L) or TST Expert and TST Local (R); ICC(A,1) = Intraclass correlation coefficient of type (A,1); LLA = lower limit of agreement.

Figure 6 shows the Bland–Altman and correlation analysis for the TST estimate. A significantly lower correlation was found for PAT HSAT as well as a wider distance between the limits of agreement.

## Discussion

### Endpoint analysis highlights and discussion

We found that, for both the 3% and the 4% Rule, most primary endpoint parameters showed a close agreement with PSG. The disparity between PAT HSAT and the Local Analysis’ performance was the smallest for the 4% Rule. This is unsurprising since the 4% Rule differs from the 3% Rule in that the latter does not consider arousals. The scoring of cortical arousals suffers from significant inter-scorer variability [16], trickling through to a larger inter-scorer variability for hypopnea scoring. The NPV at AHI cutoff 5 of 62% for PAT HSAT and 73% for the Local PSG Analysis (using 3% Rule) highlights a tendency of both to underscore OSA. However, only one patient classified as negative by PAT HSAT or the Local Analysis was diagnosed with moderate OSA by the Expert Analysis, therefore supporting the conclusion that it is unlikely to misdiagnose a patient with moderate OSA as having no OSA. These findings contrast with recent findings of Zhang et al. [17] which reported a strong overscoring of mild OSA by WatchPAT with a specificity at AHI cutoff 5 of only 29%.

The 4-way categorization accuracy of 70% (3% rule) and 78% (4% rule) of PAT HSAT was significantly stronger than the 4-way categorization accuracy of 61% reported by the most recent large-cohort manufacturer-sponsored validation study of WatchPAT, or the 53% accuracy reported by the largest independent validation study [7]. A significantly lower ICC(A,1) as well as significantly wider LoA interval was found for the total sleep time estimate of PAT HSAT compared to the Local PSG Analysis. This confirms the previously reported underperformance of PAT HSAT compared to PSG [8, 17] in estimating sleep time, which is unsurprising as PAT HSAT makes use of actigraphy which is merely an approximation of true sleep time as estimated by EEG. Therefore, PAT HSAT cannot be considered a valid substitute for PSG as it pertains to the assessment of sleep (stages).

Finally, the strong increase in most endpoint parameter values when applying NBL further illustrates the need to adequately handle near-boundary AHIs. These findings warrant further discussion on whether patients with an AHI in NBZ would benefit from further diagnostic evaluation to increase confidence in therapy decision making.

### Strengths and limitations of study

A first strength of this study is its adequately powered multicentric design, incorporating centers located in Europe as well as the United States. A second strength is its unique approach in double labeling of PSG so as to allow for the comparison of the agreement of PAT HSAT with PSG to the inter-rater agreement of PSG. A third strength lies in its critical assessment of clinical endpoint parameters, including only endpoint parameters which serve the purpose of assessing whether the device can safely and effectively help navigate the patient through the diagnostic pathway. A fourth strength of the study is its unique approach to dealing with spurious endpoint parameter variability caused by reference AHIs close to OSA severity boundaries.

A limitation of the study is the lack of assessment of the impact of multi-night testing on the endpoint parameters, as PAT HSAT is typically administered for multiple nights. Inter-night variability has been shown to be a significant contributor to diagnostic errors [18].

## Conclusion

This multicentric validation study of the PAT HSAT was designed to robustly assess whether the device can adequately navigate the patient through the diagnostic pathway, i.e. whether it can adequately determine the OSA severity.

The unique cornerstones of its design are the double-labeling of PSG so as to establish a performance target for HSAT, adequate treatment of AHIs close to the OSA severity category boundaries, and the avoidance of reliance on misleading clinical endpoint parameters such as Pearson and Spearman correlation coefficients [15]. Replication of these design cornerstones increases transparency of clinical endpoints and can enable more generalization and standardization of future HSAT validation studies.

For both the 3% and the 4% Rules, most endpoint parameters showed a close agreement with PSG when compared with the inter-rater variability of the PSG. The 4-way categorization accuracy of PAT HSAT was strong, in particular in comparison to reported performances of similar HSATs, but nevertheless lower than the inter-rater agreement of PSG (70% vs 77% for the 3% Rule and 78% vs 81% for the 4% Rule).

## Data Availability

The data that support the findings of this study are available from the corresponding author upon reasonable request.

## Notes

Clinical Trials

NCT04191668, A Validation Study of the NightOwl PAT-based Home Sleep Apnea Test, https://clinicaltrials.gov/ct2/show/NCT04191668
