# Correction to: A multicentric validation study of a novel home sleep apnea test based on peripheral arterial tonometry

**DOI:** 10.1093/sleep/zsaf187

**Published:** 2025-07-15

This is a correction to: Bart Van Pee, Frederik Massie, Steven Vits, Pauline Dreesen, Susie Klerkx, Jagdeep Bijwadia, Johan Verbraecken, Jeroen Bergmann, A multicentric validation study of a novel home sleep apnea test based on peripheral arterial tonometry, *Sleep*, Volume 45, Issue 5, May 2022, zsac028, https://doi.org/10.1093/sleep/zsac028

The published version of this manuscript omitted to note that Bart Van Pee, Frederik Massie and Steven Vits contributed equally to this manuscript and are to be considered joint first authors.

These details have been corrected only in this correction notice to preserve the published version of record.

